# “A prática da inoculação da vacina e as suas funestas consequências”: uma obra contra a vacina antivariólica no Império português no início do XIX

**DOI:** 10.1590/S0104-59702026000100037

**Published:** 2026-10-26

**Authors:** Fillipe dos Santos Portugal, Maria Rachel Fróes da Fonseca

**Affiliations:** i Programa de Pós-graduação em História das Ciências e da Saúde, Casa de Oswaldo Cruz, Fundação Oswaldo Cruz, Fiocruz. Rio de Janeiro – RJ – Brasil. fillipe_portugal@hotmail.com; ii Departamento de Pesquisa, Casa de Oswaldo Cruz, Fundação Oswaldo Cruz, Fiocruz. Rio de Janeiro – RJ – Brasil. rachel.froes@fiocruz.br

**Keywords:** Vacina, Varíola, Portugal, Heliodoro Jacinto de Araújo Carneiro (1776-1849), Vaccine, Smallpox, Portugal, Heliodoro Jacinto de Araújo Carneiro (1776-1849)

## Abstract

Este artigo analisa os principais debates sobre a vacina antivariólica no Império português, no início do século XIX, com ênfase na obra *Reflexões e observações sobre a prática da inoculação da vacina e as suas funestas consequências* (1808), do médico português Heliodoro Carneiro. Trata-se de uma das primeiras obras publicadas em Portugal sobre a vacina antivariólica, destacando-se por sua posição contrária à vacinação. A análise da obra é relevante por integrar perspectivas médicas, sociais e científicas que embasaram a recusa à vacina, fomentando debates e contestações por parte de diversos médicos. Assim, este estudo contribui para o aprofundamento dos debates sobre a vacina e a medicina no século XIX.

## Abstract (en)

This article analyses the main debates on the anti-smallpox vaccine in the Portuguese Empire in the early-nineteenth century, emphasizing the work Reflections and observations on the practice of vaccine inoculation and its dire consequences (1808), by the Portuguese physicist Heliodoro Carneiro. It was one of the early works published in Portugal on the anti-smallpox vaccine, highlighting his position against the vaccination. The analysis of the work is relevant because it integrates medical, social and scientific perspectives that substantiated the refusal to the vaccine, fomenting debates and objections from other physicists. Thus, this study contributes to deepen the debates on vaccine and medicine in the nineteenth century.

A vacina antivariólica acabou se tornando um sucesso científico, vide a erradicação da ocorrência da doença entre os seres humanos no século XX. Todavia, no contexto de sua descoberta e difusão, não havia explicações teóricas suficientes para explicar seu efeito protetivo, e vários médicos se manifestaram contrários à prática, fundamentando-se em argumentos científicos, como também de natureza política e cultural. No século XIX, o principal representante desse grupo de médicos no Império português foi Heliodoro Jacinto de Araújo Carneiro (1776-1849), autor da obra *Reflexões, e observações sobre a prática da inoculação da vacina, e suas funestas consequências: feitas em Inglaterra*, publicada em 1808.

O livro de Heliodoro Carneiro teve grande repercussão, despertando fortes reações no meio médico lusitano. Alguns dos nomes mais influentes da medicina portuguesa da época apresentaram objeções contundentes à obra, posicionando-se firmemente em defesa da prática da vacinação. Essa resposta ganhou visibilidade especialmente por meio da publicação de duas extensas matérias no periódico *O Investigador Português em Inglaterra ou Jornal literário, político etc.*, em 1811 e 1812. Essas matérias traziam o relato de uma gama de médicos portugueses sobre a vacina, e não apenas buscavam refutar os argumentos contrários apresentados por Carneiro, como também recomendavam a leitura de autores britânicos favoráveis à vacinação, em um esforço para confrontar diretamente os pensadores ingleses citados por ele. Tal mobilização demonstra a intensidade do debate em torno da vacinação dentro do Império português e indica o grau de inserção das elites intelectuais lusas nas redes de circulação de saberes científicos europeus.

As obras mencionadas constituem as principais fontes deste artigo. A partir delas, realizaremos uma análise fundamentada na metodologia da história das ciências, em especial no princípio da simetria, conforme proposto por Bloor (1976). Tal princípio parte da premissa de que acertos e erros científicos devem ser analisados com os mesmos critérios de cientificidade, sem pressupor que o conhecimento atualmente aceito seja intrinsecamente superior ao anteriormente rejeitado. As teorias científicas não ganham legitimidade apenas por oferecerem explicações mais verdadeiras ou fidedignas da realidade, mas porque são sustentadas por causas sociais, como disputas institucionais, redes de credibilidade e contextos históricos, que conferem autoridade e reconhecimento a determinados saberes em detrimento de outros.

Assim, ao longo dos anos, a vacina antivariólica foi sendo cada vez mais aceita pela comunidade médica, em razão de sua eficácia. A obra de Heliodoro Carneiro, por seu turno, foi desacreditada e passou para a história como um erro científico, a ponto de seus contemporâneos apelidarem o médico de “Doutor Bexigas”. Todavia, a partir da perspectiva metodológica adotada, compreendemos que os argumentos contrários à vacina apresentados por Carneiro eram plausíveis para o campo teórico da medicina de sua época.

O estudo da história da vacina antivariólica no Brasil tem como marcos importantes as obras de Tania Fernandes (2010) e Sidney Chalhoub (1996), que resgataram as dimensões históricas dessa prática em meio a disputas científicas e a distintas conjunturas político-governamentais. Atualmente, a história da vacina vem sendo revisitada por historiadores, que têm encontrado novas fontes e percorrido novos caminhos argumentativos. Destacam-se obras que analisam a introdução e promoção da vacina para além do eixo Rio-São Paulo, como Hochman e Souza (2022), sobre a vacinação na Bahia; o estudo comparado de Brizola (2024), sobre a vacina nas cidades de Havana e Rio de Janeiro; ou ainda aquelas que traçam paralelos entre os negacionismos em torno da vacina antivariólica e da vacina contra a covid-19, como discutem Fernandes e Pinheiro (2021).

Nosso artigo busca contribuir para esse campo ao focalizar os debates em torno da aceitação da vacina no contexto de sua introdução, aprofundando a análise da diversidade de argumentos, tanto favoráveis quanto contrários, que mobilizaram uma ampla gama de atores, incluindo médicos, políticos e instituições. Ao examinar essas controvérsias, procuramos evidenciar como o processo de legitimação da vacina ocorreu em um terreno permeado por tensões ideológicas, interesses científicos e dinâmicas sociais.

O estudo histórico sobre as vacinas é um debate atual, extremamente relevante e instigante. Segundo Hochman et al. (2002), o estudo sobre a história da medicina é essencial tanto para a história social quanto para a saúde pública no Brasil, pois contribui para a compreensão da origem e complexidade dos processos, bem como para o enfrentamento de desafios da saúde coletiva, ou mesmo a implantação de políticas de saúde.

Segundo Anne Moulin (2003), as vacinas seriam “um gigante em pés de barro”. Para a historiadora francesa, o adjetivo “gigante” qualifica a vacina como uma das práticas mais contundentes no combate às enfermidades. Os “pés de barro”, por seu turno, seriam em função da forte oposição social às diferentes vacinas, que, desde sua descoberta, têm sua utilidade frequentemente questionada, tornando-se até mesmo alvo de revoltas populares. Esse fenômeno pode ser observado também nos dias atuais, especialmente durante a pandemia de covid-19, quando surgiu um intenso debate em torno da criação das vacinas contra o novo coronavírus. Nesse período, registrou-se, em nível mundial, uma queda nas taxas de vacinação contra diversas outras doenças (Abreu et al., 2022).

O texto está dividido em três seções. Na primeira, “Notas sobre a vacina antivariólica e sua introdução em Portugal”, apresentamos e discutimos a conjuntura em que a vacina foi introduzida nos domínios portugueses. Na segunda seção, “O ‘Doutor Bexigas’: uma obra contra ‘o bestial’ projeto da vacina”, examinamos a obra de Heliodoro Carneiro e seus argumentos científicos e socioculturais contra a vacina antivariólica. Na terceira e última parte, “Heliodoro Carneiro se rende à vacina: respostas às suas objeções”, apresentamos as respostas dos médicos portugueses contra os argumentos de Heliodoro Carneiro especialmente a partir do periódico *O Investigador Português em Inglaterra*.

## Notas sobre a vacina antivariólica e sua introdução em Portugal

A varíola, atualmente, é considerada uma infecção viral contagiosa, causada pelo vírus *Poxvirus variolae*, que pertence a um grupo de vírus (grupo varíola-vacínia) (Gazêta, 2006, p.27). Essa doença causa o surgimento de diversas erupções de vesículas pustulosas que podem deixar diversas cicatrizes no corpo, ocasionando cegueira e até mesmo a morte. Por não necessitar de vetores intermediários, a varíola se espalhou rapidamente e se tornou uma das doenças que mais mortes causaram na história da humanidade (Martins, 1997, p.125).

O dito popular, que circulou na Alemanha do século XVIII, de que “da varíola e do amor são poucos que conseguem escapar” [*Von Pocken und Liebe bleiben nur Wenig frei*] é uma evidência da dificuldade de uma pessoa, na Europa naquela época, passar pela vida sem ser acometido pela varíola (Morris, 1896, p.1). Aqueles que não tiveram a doença viviam sob constante medo de contraí-la, e os que sobreviviam carregavam consigo as marcas por ela deixadas, como a cegueira e diversas cicatrizes, principalmente na face (Pereira, 2007, p.95). Esse fato se refletia na obra de diversos médicos europeus da época, que consideravam desnecessária a descrição da doença, dada a frequência com que ocorria.

Segundo a pesquisadora Tania Fernandes (2010), a constatação da transmissibilidade e da proteção da varíola, que são características dessa doença, impulsionou a disseminação de diversas técnicas que buscavam evitar sua forma mais grave a partir da introdução, no homem sadio, do vírus variólico contido na secreção retirada da pústula de pessoas doentes. Essas práticas receberam o nome de inoculação ou “variolização” (p.16). A inoculação ocorreu, embora sem grande difusão, em diversas partes do mundo. Apesar da utilidade, a prática trazia o risco de provocar nas pessoas inoculadas diferentes manifestações da moléstia, chegando muitas vezes a ocorrer a forma mais grave da varíola, que levava o indivíduo à morte. Devido a esse fator, a “variolização” ao longo do tempo foi objeto de grandes controvérsias quanto à sua segurança e eficácia, pois acreditava-se que sua prática, em meio a uma epidemia, poderia agravá-la seriamente (Chalhoub, 1996, p.104).

Em 1798, o inglês Edward Jenner descobriu a vacina antivariólica, e daí se tornou comum chamá-la de vacina jenneriana. A vacina contra a varíola era produzida a partir de uma doença que acometia o gado bovino, conhecida como *cow-pox*. Jenner obteve a vacina pela observação de que pessoas que trabalhavam com o gado e contraíam o *cow-pox*, consequentemente, não adquiriam a varíola. Em contrapartida, não havia referenciais teóricos para explicar o processo imunizante. Como não era uma doença muito comum no gado, acabava-se por utilizar o método de vacinação “braço a braço”, retirando-se o fluído vacínico de uma pessoa já vacinada e aplicando direto no braço de outro indivíduo. Nessa conjuntura, diversas dúvidas surgiram quanto à eficácia da vacina e à possibilidade de transmissão de outras doenças por meio de sua utilização (Fernandes, 2010, p.31-32).

A vacina jenneriana, no entanto, rapidamente lograria grande êxito, pelo fato de a “variolização” conter maiores riscos de contágio, uma vez que as pessoas que adquiriam a varíola de vaca não adquiriam a varíola humana, como ocorria no processo de inoculação. Jenner sabia que a doença das vacas, embora produzisse feridas nas pessoas, não era tão grave quanto a varíola e nunca era letal (Chalhoub, 1996, p.107). Em estudos anteriores, destacamos que diversos periódicos europeus noticiaram a eficácia do método vacinal, especialmente ao relacioná-lo com o aumento populacional registrado em diversas cidades do mundo, vinculado à prática da vacinação (Portugal, 2018).

No contexto português, a vacina surgiu em meio à conjuntura do Antigo Regime, período em que a saúde pública se baseava em dois paradigmas distintos. O primeiro sustentava que cabia ao príncipe zelar pela saúde de seus vassalos como se fossem membros de sua própria família, em um modelo pautado pela lógica familiar e pela pastoral cristã. No segundo, a população passa a ser vista como um recurso valioso para a construção de um Estado próspero e desenvolvido, o que leva à criação de políticas voltadas para sua ampliação e preservação (Subtil, 2015).

Assim, o Estado português, desde o final do século XVIII, inicia uma política voltada para a redução da mortalidade e a promoção do crescimento populacional. Para isso, o governo adotou uma série de medidas, como o estímulo aos matrimônios, a promoção da natalidade, a prevenção e o controle de doenças venéreas, mas, principalmente, o combate à varíola por meio do método da inoculação e, posteriormente, da vacinação (Barreiros, 2014). Dessa forma, em 1799, uma carta régia foi enviada a todos os governadores dos domínios ultramarinos com o objetivo de possibilitar a introdução da inoculação contra as bexigas, especialmente entre a população de meninos negros e indígenas. Entendia-se que essa prática era “o único meio eficaz contra as bexigas naturais” (Portugal, 2018, p.37), que vinham causando grandes prejuízos ao reino. Já em 1802, novas cartas régias foram expedidas, incentivando a disseminação da vacina jenneriana por todas as capitanias do Império (p.37).

José Silva (2015) observa que a vacinação em Portugal teria começado como uma prática localizada e privada, e aplicada de modo não sistemático. No grupo de vacinadores estavam pessoas de diferentes perfis, desde médicos e cirurgiões até pessoas não especializadas como padres, sangradores e pessoas movidas por ideais filantrópicos ou humanitários. Assim, a distribuição da vacina teria ficado restrita a poucas áreas de Portugal, e teria se formado uma “malha bastante rarefeita e pouco eficaz em termos de cobertura do território” (Silva, 2015, p.272-273). No Brasil, a introdução da vacina se deu por comerciantes baianos, que importaram material varioloso da Europa e demonstraram interesse em vacinar a escravaria, vista como foco de contágio. Relatos da época também mencionam as grandes perdas financeiras causadas pela morte de escravizados, trazidos da África para sustentar o trabalho nos domínios portugueses na América (Brizola, 2024). Além disso, a manutenção da vacina esteve diretamente ligada à ação governamental, tendo ocorrido de maneira mais contundente na Bahia e no Rio de Janeiro (Portugal, 2018).

O fato é que a querela sobre a vacina já estava posta nos domínios portugueses no começo do século, quando o governo português encomendou a Manuel Henriques de Paiva (1752-1829), médico e boticário no Hospital Real Militar e Ultramar, a produção de uma obra sobre o tema. Assim, em 1801, Henriques de Paiva publicou a obra *Preservativo das bexigas e de seus terríveis estragos ou história da origem e descobrimento da vacina, dos seus efeitos ou sintomas, e do método de fazer a vacinação*.^1^ Segundo o historiador João Rui Pita (2009), a intenção da obra era a de alertar a população para a varíola como um grande problema de saúde, de modo a sensibilizá-la a aderir a um processo “garantido e inovador”, que era a vacina.

Em sua obra, Henriques de Paiva (1801) buscou responder aos principais questionamentos existentes sobre a vacina, como a existência de poucos estudos sobre o tema e a questão de que não se devia introduzir no gênero humano uma doença nova trazida pelos animais. Como resposta a esses questionamentos, Henriques de Paiva argumentou que a prática da vacinação estava amplamente disseminada por toda a Europa, que o método era seguro e que não haveria problema em introduzir uma “doença inocente” que poderia livrar a população de outra tão funesta como a varíola. Dessa maneira, Paiva recomendava que os médicos publicassem suas observações e incentivava que a população deixasse de lado seus “prejuízos” contra a vacina.

Embora em Portugal existissem muitas posições favoráveis à prática da vacinação, não havia unanimidade sobre o assunto. Isso fica evidente no episódio envolvendo o médico Ignácio Tamagine, que, ao tentar introduzir a vacina na vila de Abrantes, em 1803, foi impedido pelo juiz de fora. O magistrado alegou que as autoridades ainda divergiam quanto à adoção daquele método, então considerado novo. A controvérsia chegou às mãos do intendente geral da Polícia, que se viu indeciso diante de duas publicações oficiais com posições opostas – uma a favor e outra contra a vacina. Diante disso, encaminhou o caso à avaliação do corpo acadêmico da Faculdade de Medicina da Universidade de Coimbra. Também cabia uma análise do protomedicato, cujo parecer, emitido ainda em 1804, manifestou-se favoravelmente à vacinação, autorizando, assim, sua prática por Ignácio Tamagine e por outros médicos que desejassem empregar o método (Barreiros, 2014, p.218-219).

Fica, então, evidente que, a despeito dessas notícias positivas, já circulavam, em Portugal, desde o início de sua prática, diversas obras contra a vacinação. Um fato favorável para a disseminação da vacina ocorreu em 1804, quando o príncipe-regente dom João, “convencido da utilidade da vacina”, havia mandado vacinar os seus filhos. O exemplo era importante, pois a vacinação dos filhos do monarca podia influenciar uma maior aceitação da vacina, e marcar derradeiramente o que seria uma política absolutista em favor da vacinação (Provimentos militares, 9 ago. 1805).

## O “Doutor Bexigas”: uma obra contra “o bestial” projeto da vacina

Esta obra é tal que o seu Autor parece ter tido em a fazer o mesmo intuito de Horostrato quando queimou o templo de Diana em Éfeso; isto é quis fazer-se celebre por uma singularidade, pela qual merecia sofrer realmente a mesma pena, que se impôs ao seu protótipo (Gomes, 1812, p.23).

Ao comentar sobre a obra *Reflexões e observações sobre a prática da inoculação da vacina, e as suas funestas consequências feitas em Inglaterra*, de Heliodoro Jacinto de Araújo Carneiro (1776-1849),^2^ o médico e botânico português Bernardino António Gomes (1768-1823) apresentou uma analogia com a trajetória de Heróstrato, no Templo de Diana, considerado uma das sete maravilhas do mundo antigo. Heróstrato era um habitante desconhecido da cidade de Éfeso que havia procurado tornar-se célebre queimando o sobredito templo e, por causa do incêndio provocado, fora condenado à morte e seu nome proibido de ser pronunciado sob pena de morte. Essa analogia representava o impacto da obra de Heliodoro Carneiro contra a vacina e a posição dos médicos pró-vacinação, que o criticaram por gerações e que lhe haviam conferido a alcunha de “Doutor Bexigas”, pois ao detratar a vacina acabava por favorecer a varíola (Gomes, 1812, p.23).

A obra polêmica de Heliodoro Jacinto de Araújo Carneiro surgiu no contexto do interesse crescente do governo português pela prática da vacinação. O médico teria sido nomeado para realizar um “curso de viagens e observações médicas pelos diferentes países da Europa” (Carneiro, 1808), do qual resultou a publicação de sua obra, em 1808, considerada a crítica mais contundente à vacina antivariólica nos domínios portugueses.

Para construir a sua obra, Heliodoro Carneiro não praticou efetivamente a vacinação, mas se baseou nas obras de Jenner e de outros autores, além de ter visitado os práticos e os hospitais particulares na Inglaterra. Dessa maneira, argumentou ter entrado em contato com todos os fenômenos em relação à vacina e suas “funestas consequências”. Sua argumentação foi toda pautada contra a vacina, e para isso utilizou argumentos que circundavam os campos médico, social, cultural, teórico, histórico e científico. Para o autor, a utilização do material vacinal extraído das vacas era uma forma de desonra ao animal, que fornecia importantes alimentos à humanidade como carne, leite e derivados. Mas, em contrapartida, “era paga com indignidade e degradação de sua espécie” (Carneiro, 1808, p.11).

O pensamento de Heliodoro Carneiro está inserido nos ideais neo-hipocráticos da medicina, na compreensão de que as doenças não tinham uma causa única, mas eram pluricausais. Seguindo os ideais de Hipócrates e Galeno, as doenças ocorriam devido ao desequilíbrio dos humores encontrados no corpo, atrelados a influências externas que iam desde as forças da natureza, dieta e alimentação, até a influência dos astros e do clima. Nessa perspectiva, a ideia ampliada de clima incluía tanto a temperatura e diferentes aspectos topográficos quanto os movimentos das estrelas, cometas e planetas, além da própria vegetação e de outros aspectos. A forma comum de curar as doenças estava ligada à ideia de trazer de volta esse equilíbrio, por meio de diversos tratamentos presentes na medicina da época, como as sangrias e purgantes (Abreu, Nogueira, Kury, 2018, p.30).

Nesse contexto, Carneiro (1808, p.ii) acreditava que a vacina era causada e produzida por “variações e constituições da atmosfera”, por meio de uma “matéria morbosa” originada da vaca, animal que teria a “natureza” muito diferente da do homem. Assim, para Carneiro (1808), a vacina ia diretamente contra o paradigma médico vigente, segundo o qual não se poderia passar uma doença da “economia animal” para a humana sem provocar uma série de consequências indesejáveis; entre elas, o surgimento de novas doenças no organismo humano. Por isso, para Heliodoro Carneiro (1808, p.53), o objetivo de sua obra estava circunscrito em demonstrar como a vacina estava fazendo vítimas cotidianamente devido à “ignorância de uns e obstinacidade [*sic*] de outros”.

Especificamente sobre a estrutura da obra de Heliodoro Carneiro, observamos que, num primeiro momento, o autor destacou o histórico da inoculação de bexigas e sua eficácia na Europa do século XVIII. Em sua opinião, seria mais racional evitar a varíola por meio de mais pesquisas e melhoramento da prática da inoculação, como já vinha sendo feito há mais de um século na Europa, do que tentar evitar uma doença da “raça humana” por outra tão diferente, como a dos quadrúpedes.

Nesse ponto, o autor criticava o governo inglês, que havia proibido a inoculação e a substituído pela vacinação, com o agravante de que a prática médica se dava mesclada aos deveres eclesiásticos, ao ordenarem que os bispos e dioceses pregassem e intimassem seus pupilos a se vacinar. Com isso, na visão de Heliodoro Carneiro (1808, p.5), a vacina “gerou o zelo e crime” de muitos padres e médicos, que estavam fazendo com que “um veneno que atacava e flagelava a raça humana se fazia por familiarizar com a economia animal em lugar de expulsá-lo e destruir”. Ademais, para Heliodoro Carneiro, a cultura inglesa havia favorecido a vacina, pois tinham uma “grande afinidade” com a vaca, animal que tinha raízes em seus primeiros antepassados, como John Bull:^3^ “Um homem indômito, forte e bravo com um touro” (Carneiro, 1808, p.50).

Como estratégia de argumentação, Heliodoro Carneiro fez ataques pontuais à obra de Edward Jenner, referindo-se diretamente a trechos de suas publicações. Em oposição ao fato de Jenner amenizar os sintomas da vacina, Heliodoro Carneiro buscava realçá-los, descrevendo calafrios, cefaleia, dores lombares e nos membros. Para Heliodoro Carneiro, como Jenner não podia negar a gravidade desses sintomas, recorreu ao argumento de que eles decorriam da irritação da úlcera, e não da ação do vírus vacinal sobre a constituição humana, um subterfúgio que, segundo Carneiro (1808, p.21), era “pouco ou nada feliz”.

Heliodoro Carneiro também criticou o rigor científico da obra de Jenner, pois “os erros em sua análise seriam visíveis por toda a obra”, tanto nas questões teóricas quanto nas práticas. Assim, Carneiro questionou, ao longo de sua obra, o fato de Jenner apresentar as observações dos “paisanos de Gloucesteshire”, que estariam fundadas na “tradição e contos”, como fatos comprobatórios da vacina, pois tais não seriam válidas como evidências científicas. Entendia que esses leigos não sabiam diferenciar os diferentes estágios da própria varíola e nem atestar sua “proteção” contra a doença. Carneiro (1808, p.23) afirmou que as pessoas poderiam ter tido bexigas, muitas vezes com pouca, ou nenhuma erupção, e só febre, assim “como podiam os paisanos de Gloucestershire conhecer, e decidir se as pústulas originadas nas vacas livrarão, ou não, das bexigas?”.

Em relação à teoria de Jenner para alicerçar a vacina, Heliodoro Carneiro não aceitava que a origem da vacina estava, como Jenner defendia, em uma doença que teria surgido nos cavalos e mudado sua constituição ao passar para as vacas e depois para o homem. Além disso, para Carneiro, a ideia de Jenner de que as bexigas fossem a vacina, porém “mais exaltada e forte”, podia ser desacreditada, pois não fazia sentido que a bexiga, que era mais forte, não pudesse evitar que uma pessoa adquirisse a vacina, e que não haveria sentido que ela conseguisse proteger o “sistema” contra as bexigas. Além da implausibilidade teórica, Heliodoro Carneiro ressaltou que essa teoria carecia de teste empírico para comprová-la, pois Jenner não teria se baseado em experiência direta, mas sim em “alguns contos populares” (Carneiro, 1808, p.29-30).

Ainda no campo das críticas teóricas, Heliodoro Carneiro baseia-se na obra *Treatise on the lues bovilla, or cow-pox*, publicada em 1805, pelo inglês Benjamin Moseley (1742-1819), médico do Royal Hospital Chelsea e uma das vozes mais influentes contrárias à vacinação no início do século XIX. Alguns dos principais argumentos presentes na obra são reproduzidos por Heliodoro Carneiro (1808, p.60):

Por que diz ele, pelos princípios de patologia e boa analogia, das leis da economia animal, e da falta de semelhança entre as duas moléstias, é impossível crer sem uma inteira alienação da razão, que qualquer delas possa tornar a constituição insuscetível da outra. As bexigas não têm afinidade alguma com a vacina, mas com outra qualquer moléstia, e as bexigas somente podem ser mencionadas com a vacina para significar a sua mútua dessemelhança.

Os argumentos de Benjamin Mosely seriam plausíveis, pois, como Tania Fernandes (2010) aponta, na época de Jenner não havia referenciais teóricos que possibilitassem a compreensão do processo imunitário ou que indicassem hipóteses a serem comprovadas empiricamente. Para a autora, o próprio Jenner, em suas obras, não tentou explicar como ocorria o fenômeno de “proteção” que incidia após a vacinação, contentando-se apenas em afirmar que a vacina funcionava e era segura.

Heliodoro Carneiro também ressaltou o problema de passar doenças dos animais para os seres humanos. Segundo o facultativo, cada reino, o mineral, o vegetal e o animal, possuiria suas próprias “eflorências e excrecências morbosas”, cada qual com leis gerais e comuns, mas também com leis particulares e próprias a cada classe, ordem e espécie. Seguindo esses princípios, a introdução do pus da vaca não faria alterações no sistema orgânico do homem, por ser um “pus bestial”, de modo que o que causava a defesa do corpo contra a varíola ocorria em todos os casos em que o “sistema” fosse atacado por uma doença, e dessa forma ficava temporariamente não susceptível a outra, como nos casos em que as pessoas com sarna não pegavam “lepra”. Com isso, Heliodoro Carneiro (1808, p.33-37) citou diversos exemplos, buscando ratificar que a capacidade protetiva da vacina seria uma qualidade comum a outras moléstias, e que as suas consequências seriam demasiado graves para serem utilizadas como um preservativo.

Procurando fundamentar seu argumento, Heliodoro Carneiro mencionou os exemplos das transfusões de sangue do animal para o homem, realizadas no século XVII. Segundo Heliodoro Carneiro, esse procedimento estava baseado na crença de que, pela transfusão de sangue, poderiam ser transmitidas características dos animais para o homem e, assim, curar seus caracteres viciosos, como, por exemplo, o sangue do leão curaria a covardia, e o da ovelha, a ferocidade. Para o médico, na época de sua criação, a técnica parecia promissora, e rapidamente se espalhou a crença de que poderia livrar o ser humano de todas as doenças, sendo diversos relatos publicados na época incentivando sua prática. Entretanto, as pessoas que aderiam à novidade logo que recebiam a transfusão se tornavam inválidas, doentes, loucas e depois sucumbiam à morte, e, assim, a prática acabou sendo proibida pelo parlamento inglês em 1670 (Carneiro, 1808, p.52-54).

Outro aspecto interessante abordado na obra de Heliodoro Carneiro foi a associação entre os efeitos da vacina e as condições climáticas. Conforme afirmamos anteriormente, Heliodoro Carneiro seguia o paradigma neo-hipocrático, segundo o qual o calor e a umidade eram vistos como fatores que produziam efeitos indesejáveis nos corpos e na saúde dos indivíduos, tanto no aspecto físico quanto no moral. Associava-se esse clima aos trópicos, que eram, assim, transformados em um local de doenças (Caponi, 2007, p.964). Assim, para o autor, a vacina aplicada em regiões de climas quentes e temperados desenvolveria mais erupções do que quando aplicada em locais de climas “frios e irregulares”. Tal situação ocorreria em razão da maior e mais regular transpiração nas regiões de climas quentes, onde as “funestas consequências” da vacina seriam ainda mais alarmantes. A “esses climas” se somariam as “mudanças da atmosfera”, e/ou incidentes devido ao “regime e vida do vacinado”, que poderiam ocasionar maior “disposição bestial” da vacina e maior ocorrência de febres e desordens do pior caráter. Com isso, o autor teoriza que, nas colônias, embora os negros vacinados tivessem mais propensão à forma mais grave da doença, e talvez muitos morressem, assim como ocorrera na Inglaterra, suas mortes seriam atribuídas às “más constituições e irregularidades das estações das colônias” (Carneiro, 1808, p.110-111).

Passando do campo teórico para os casos empíricos, Heliodoro Carneiro constatou que em pessoas vacinadas seria possível observar desordens e moléstias difíceis de curar, haja vista que algumas adquiriam doenças que atacavam a maior parte do gado, como a sarna, a lepra dos cavalos. Nesses animais existiria uma espécie de “constituição pituitosa” que gerava tumores, que eram análogos aos observados em pessoas vacinadas. Em suas palavras, as pessoas vacinadas estariam adquirindo “moléstias bestiais, as quais nunca antes a espécie humana havia experimentado”. Além disso, para alguns médicos, a vacina causava nas crianças “uma diminuição da viveza de espírito”. Em um caso extremo, relatado pelo médico escocês John Ferdinand Smyth Stuart (1745-1814), uma criança, após ser vacinada, “perdera a fisionomia humana” (Carneiro, 1808, p.89-95).

A vacina também seria seguida de erupções que trariam complicações, chegando a comprometer o sono das crianças e tornando sua cura “difícil e rebelde”. Poderiam também surgir ulcerações e gangrena nos braços e, em alguns casos, a perda do nariz e das extremidades dos dedos. Por todos esses reveses, na visão de Heliodoro Carneiro, a vacina era um empreendimento fadado ao fim, pois, quanto mais os vacinadores fossem ativos em propagar o “bestial projeto”, mais depressa a humanidade seria convencida de seus malefícios. Para Heliodoro Carneiro, os malefícios da vacina seriam imprevisíveis, como sinalizara o médico britânico Benjamim Moseley, frequentemente citado por Carneiro (1808, p.94): “Poderá alguém predizer que poderão ser as consequências de introduzir um humor bestial na constituição humana passados muitos anos? ... Quem sabe igualmente o quanto o caráter humano poderá sofrer de estranhas mudanças de simpatia quadrúpede”.

Heliodoro Carneiro afirmou que, no ano de 1806, a vacina já havia mostrado, em Portugal, efeitos funestos análogos àqueles ocorridos na Inglaterra. Segundo o autor, muitas crianças em Lisboa que tinham sido vacinadas estavam sofrendo com erupções e febre, a que alguns médicos davam o nome de “sarampão”, embora não tivesse nenhum sintoma próprio ao sarampo. A doença estava sendo tão recorrente que algumas crianças chegaram a ter mais de dez vezes o “sarampo” (Carneiro, 1808, p.133-134).

Outra parte importante da obra de Heliodoro Carneiro foi a apresentação de oito questionamentos contrários à vacina, acompanhados das respostas dos médicos que defendiam essa prática. Como exemplo, ao se questionar se alguma criança tinha tido bexiga ou mesmo morrido depois de ter sido vacinada, a resposta era de que a vacina utilizada não teria sido a verdadeira, mas sim a “espúria”.^4^ No caso de ocorrência de gangrena ou mortificação nos braços ou em outras partes do corpo, os médicos defensores da vacina respondiam que a gangrena e outras moléstias decorriam dos maus hábitos e de suas constituições. No caso de falecimento de um grande número de pessoas em decorrência da inoculação da vacina, respondia-se que a matéria da vacina havia sido tirada muito tarde da pústula, quando já havia uma infecção pútrida. Em último caso, quando os defensores da vacina não podiam negar que as crianças vacinadas tinham adquirido bexigas, diziam que estas seriam “bexigas volantes ou doidas”, ou seja, uma forma branda da bexiga que deveria ter sido ocasionada pelo fato de os vacinadores terem levado, por descuido, o vírus varioloso na ponta da lanceta (Carneiro, 1808, p.70-72).

Dessa forma, percebemos que Heliodoro Carneiro buscava realçar os fatores adversos associados à vacinação, enquanto as respostas dos médicos vacinadores se mostravam bastante evasivas. Nesse sentido, Carneiro (1808, p.70-72) afirmava que, quando os médicos não podiam negar que a vacinação havia de fato ocorrido e que, ainda assim, a pessoa havia contraído bexiga, eles alegavam que isso se devia ao fato de a “indentação” não ter se formado adequadamente, ou seja, a escara ou cicatriz deprimida com perda de substância que permanecia no local da inoculação.

Para Heliodoro Carneiro, embora as “funestas consequências” da vacina estivessem ocorrendo diariamente em Lisboa, tais fatos não se tornavam públicos, pois muitas das pessoas que os presenciavam eram justamente aquelas que haviam recomendado o imunizante. Além disso, muitos dos efeitos da vacina demoravam a aparecer e podiam estar associados a outros fatores. Na visão de Heliodoro Carneiro (1808, p.135): “Por isso seria preciso que todas as crianças que se inoculassem morressem imediatamente para se fazer crer que a Vacina era um mal; Ainda que para alguns não fosse bastante; como aconteceu com a morte do Duque de Miranda e da Baronesa de Quintella”.

A morte do referido duque após ter sido vacinado foi um dos fatos que mais repercutiram negativamente na prática da vacinação em Portugal, sendo utilizada pelo autor como um exemplo claro dos perigos da vacina. Outra estratégia persuasiva contra a vacina, presente na obra de Heliodoro, foi a veiculação de imagens que buscavam evidenciar a gravidade da vacinação. As ilustrações mostravam, por exemplo, úlceras provocadas tanto na vaca quanto em um menino, além de um tumor na face de uma criança vacinada, comparável às lesões observadas em porcos e vacas. Essas imagens conferiam maior força aos seus argumentos e permitiam que sua obra alcançasse o público iletrado.

**Figura 1:**
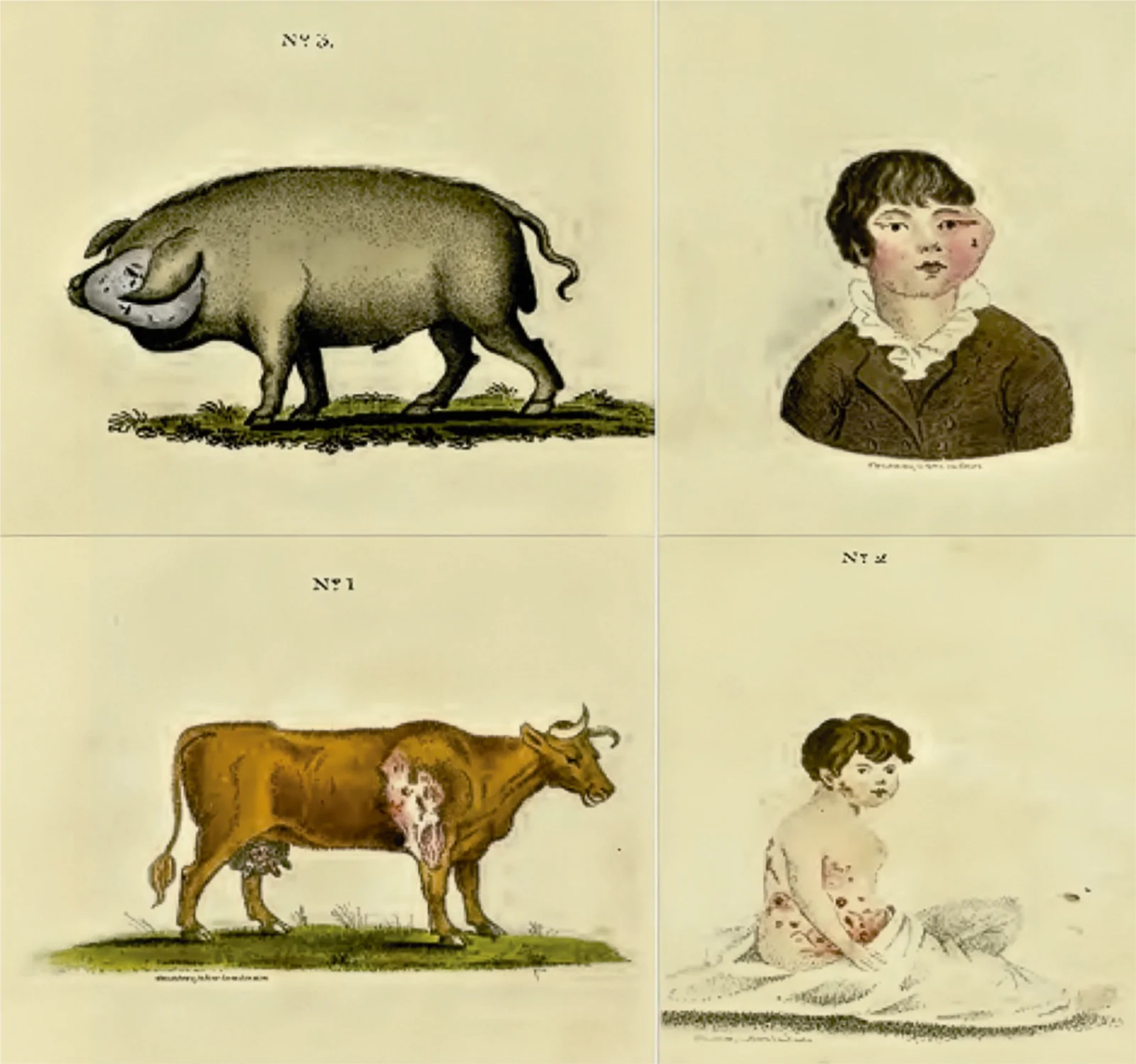
: Estampas presentes na obra de Heliodoro Carneiro que ilustram os efeitos adversos atribuídos à vacina (Carneiro, 1808, p.138-141)

Algumas manifestações contrárias à veiculação dessas figuras, na obra de Heliodoro Carneiro, apareceram em narrativas médicas portuguesas como na de Manoel Albano de Moraes, médico e cirurgião da Vila da Torre de Moncorvo que, ao prestar conta dos trabalhos em sua região, no ano de 1817, relatou que a vacinação era conhecida, porém detestada e temida entre o povo, e que por isso sua difusão era muito difícil:

Nesta época chegou às mãos de uma Mãe de família a obra de Heliodoro, a qual depois de a ler passou a outras mãos, e assim correndo até pessoas que não sabiam ler, mas que viam as quatro estampas da publicação e ficavam horrorizadas e assim fez a dita obra a sepultura de um ente tão útil, e tão proveitoso a humanidade! Sepultura coberta, carregada e seguramente fechada com as quatro estampas, que traz no fim do seu grande livro (Carta de Manoel Albano..., 1818, p.30.)

Enquanto esse médico narrava os prejuízos da disseminação de notícias contra a vacina, Heliodoro Carneiro reclamava da falta de liberdade vivenciada na imprensa, em Portugal, tendo em vista o fato de que as matérias contrárias à vacina eram censuradas e somente publicadas com “asserções e provas fictícias dos impostores e entusiastas” (Carneiro, 1808, p.51-52). Na sua visão, os médicos e boticários teriam interesse em propagar a vacina, pois ganhariam dinheiro com a aplicação do imunizante, e depois pelo tratamento dispensado aos vacinados que ficavam doentes (Carneiro, 1808).

Nesse contexto, é importante destacar que alguns dos principais periódicos da época, como a *Gazeta de Lisboa* e o *Jornal de Coimbra*, contavam, entre seus editores e proprietários, com médicos que apoiavam a vacinação. Essa condição favorecia a censura de conteúdos contrários à prática vacinal, reforçando o discurso pró-vacina nos meios de comunicação. Por outro lado, a ideia de que a vacinação pudesse ser motivada por interesses econômicos é relativizada pelo fato de que muitos de seus propagadores atuavam com motivações filantrópicas. Um exemplo disso são os vacinadores vinculados à Instituição Vacínica da Academia Real das Ciências de Lisboa, que prestavam seus serviços de forma gratuita, sem qualquer tipo de remuneração (Portugal, 2018).

## Heliodoro Carneiro se rende à vacina: respostas às suas objeções

Em meio às discussões sobre a vacina, o governo português acabou adotando uma posição favorável à prática, apoiando a criação de duas instituições destinadas à sua disseminação: a Junta da Instituição Vacínica da Corte, no Rio de Janeiro, em 1811, e a Instituição Vacínica da Academia Real das Ciências de Lisboa, em 1812 (Portugal, 2018). Segundo o então diretor da Junta da Instituição Vacínica do Rio de Janeiro, Hércules Octaviano Muzzi (1782-1841), Heliodoro Carneiro teria se rendido à eficácia da vacina:

O Dr. Heliodoro Jacinto de Araújo Carneiro chegou a publicar em Londres, em 1808, uma memória sobre a prática da vacina e suas funestas consequências: porém, convencido da força da verdade, esse, mesmo médico, bem como todos os outros que tinham abraçado sua opinião, tornou-se um dos mais zelosos sectários da vacina, e eu mesmo o vi depois vacinando aqui na casa da Instituição. Este pequeno triunfo da vacina não foi todavia sem importância, porque, derramando-se entre o povo aquele prejuízo, ele dificilmente será desarreigado (Carta de Hércules Muzzi..., 1839, p.45).

O relato de Muzzi é plausível, considerando que Heliodoro Carneiro exerceu cargos diplomáticos relevantes ao longo de sua vida, chegando a ser agraciado com o título de visconde de Condeixa. Durante o período Joanino (1808-1821), o médico realizou duas viagens ao Rio de Janeiro, buscando estreitar laços com o monarca e almejando títulos honoríficos e cargos governamentais. Mantinha, inclusive, correspondência reservada com o rei, que era padrinho de sua filha (Carvalho, 2012). Assim, alinhado com o rei, que era favorável à vacinação, faz sentido a mudança de posição de Heliodoro Carneiro.

É importante lembrar também que Heliodoro Carneiro residia em Londres como funcionário do Estado português. Na época, a capital inglesa abrigava muitos exilados lusitanos, tornando-se espaço privilegiado para o debate político e científico luso-brasileiro, em agremiações e rodas intelectuais das quais Carneiro participava. Esse ambiente favoreceu a publicação de periódicos voltados para esse público, como *Correio Braziliense, Investigador Português, O Português*, entre outros (Carvalho, 2012). Carneiro, portanto, convivia com diversos intelectuais favoráveis à vacina, que possivelmente o influenciaram. Para citar um exemplo, destacamos os redatores do *Investigador Português em Inglaterra*.

Todavia, como destacou Hércules Octaviano Muzzi, a obra de Heliodoro Carneiro teve grande impacto sobre a população. Para combater os “prejuízos” causados por suas ideias, foi publicada, três anos depois, a matéria “Reflexões e observações sobre a prática da inoculação da vacina, e suas funestas consequências, feitas em Inglaterra pelo Dr. Heliodoro Jacinto de Araújo Carneiro”, no jornal *O Investigador Português em Inglaterra,* sem identificação de autoria.^5^ O extenso texto, publicado em duas partes, foi elaborado com base em relatos de diversos médicos proeminentes e dados de vários institutos vacínicos, com o objetivo de demonstrar a eficácia da vacina e evidenciar o caráter infundado das críticas de Carneiro (Reflexões..., dez. 1811).

Para os redatores da matéria, a vacina era o tema mais discutido no campo da medicina naquela época, de modo que, se a opinião de Heliodoro Carneiro fosse verdadeira, estaria espalhando o maior bem sobre a humanidade, e, se fosse falsa, estaria fazendo o maior mal, mas, mesmo assim, este não seria culpado, pois estaria buscando a “verdade científica” (Reflexões..., dez. 1811).

Os redatores afirmavam que a matéria não se justificava pela qualidade das objeções de Carneiro, mas sim pela repercussão negativa que a sua obra causava à difusão da vacina em Portugal. Assim, na primeira parte do texto foram elencados diversos pontos relativos à obra de Heliodoro Carneiro e apresentada sua contra-argumentação.

Os méritos de Carneiro foram questionados pelos redatores, para os quais sua falta de imparcialidade certamente teria comprometido sua avaliação da vacina. Nesse sentido, Jenner, ao ter baseado suas colocações em informações de populares, teria mais validade que Heliodoro Carneiro, justamente pela imparcialidade, “visto o pouco escrúpulo, e exame com que atribui à vacina funestos efeitos, e o desejo com que diz vira, o que realmente não viu” (Reflexões..., dez. 1811, p.177).

Em relação à crítica central de Carneiro a Jenner, sobre as consequências de se inserir uma matéria bestial na constituição humana, os redatores argumentaram que, embora Heliodoro Carneiro tivesse apresentado aquelas hipóteses, não as teria provado. Segundo os redatores, Jenner conhecia tão bem quanto qualquer outro médico, por mais inculto que fosse, a teoria de que “nem todos os vírus produzem imediatamente a sua malignidade” (Reflexões..., dez. 1811, p.178). Todavia, no caso da vacina, os benefícios que ela apresentava seriam muito maiores que os possíveis riscos, fato este que seria ratificado pelo testemunho e observação constante dos habitantes de Gloucestershire (Reflexões..., dez. 1811).

Outro questionamento importante, apresentado por Heliodoro Carneiro, referia-se à visão de que as bexigas não impediam que a constituição fosse atacada pela vacina e que isto, por sua vez, demostrava que as doenças teriam pouca ou nenhuma relação. Os redatores admitiam que a bexiga, em algumas situações, não livrava contra a vacina, porém esses casos seriam raros. Todavia, para esses redatores, seguindo esses parâmetros, a inferência da falta de relação entre os vírus e as doenças seria uma conclusão precipitada, considerando que a mesma pessoa poderia contrair bexigas mais de uma vez. Desse modo, entendiam que a vacina seria um verdadeiro preservativo das bexigas, por produzir uma maior “mudança na constituição do sistema” do que as próprias bexigas. Com isso, a vacina seria capaz de extirpar a predisposição para receber o vírus varioloso, o que as próprias bexigas não conseguiriam (Reflexões..., dez. 1811).

Segundo os redatores de *O Investigador Português em Inglaterra*, embora a vacina fosse muito mais branda do que a bexiga, seria equivocado concluir que ela não pudesse livrar o sistema e deixá-lo seguro contra a doença. Haja vista que

seria necessário um conhecimento muito exato das leis da economia animal; um conhecimento perfeito da ação dos diferentes vírus sobre a mesma economia e da mudança, ou modificação que nela produzem, para poder tirar tal consequência: seria mesmo necessário conhecer, determinar em que consiste a predisposição, ou susceptibilidade de receber a ação deste ou daquele vírus. Ora o A. é mui esclarecido e ingênuo para deixar de se confessar que tudo isto se ignora, e talvez será eternamente ignorado (Reflexões..., dez. 1811, p.183).

Os argumentos presentes no referido período indicam que muitos questionamentos teóricos sobre a etiologia da vacina e da varíola estavam em aberto e não eram acessíveis ao conhecimento da medicina da época. Dessa maneira, a única forma de validar a prática e os efeitos da vacinação seria por meio dos testes empíricos e observações da vacina em todos seus estágios, como o próprio Edward Jenner havia feito. Isto é, de maneira imparcial, e não por um caminho enviesado como fizera Heliodoro Carneiro. Em relação à comparação da vacina com as práticas de transfusão de sangue, feita por Carneiro, os redatores do periódico argumentavam que a transfusão havia sido uma prática limitada à França e à Inglaterra, além de ter enfrentado forte oposição. Em contraste, a vacina alcançara proporções incomparáveis, sendo aplicada em diversos continentes e tornando-se objeto de observações e experimentações realizadas por médicos ao redor do mundo. Por isso, sustentavam que não seria possível comparar diretamente as duas práticas (Reflexões..., dez. 1811, p.183).

Os redatores de *O Investigador Português em Inglaterra*, para desacreditar Heliodoro Carneiro, haviam recorrido várias vezes ao argumento de que este nunca havia praticado a vacinação. Entendiam que, na falta de referenciais teóricos, a prática poderia ser validada pela empiria, mas Carneiro não poderia julgá-la sem praticar a vacinação, como também não conseguia observar a distinção entre a vacina verdadeira e a falsa. Essa questão, que Heliodoro Carneiro afirmara ser um subterfúgio que tornava a vacina infalível, para os autores “só poderia ser negada por quem nunca vacinou uma criança” (Reflexões..., dez. 1811, p.185). Os redatores ainda destacaram que “não aparece uma só passagem em que ele diga – eu vacinei, eu presenciei a marcha, e sintomas da vacina, em todo o tempo da sua duração etc.” (Reflexões..., dez. 1811, p.180). Em outro trecho, afirmaram enfaticamente:

É coisa bem digna de riso, ou antes bem lamentável, que um homem que nunca vacinou uma só pessoa, e que não faz mais do que jurar nas palavras de outrem, se propusesse a escrever contra a vacina! É coisa bem lastimosa que, em vez de provas reais contra a vacina, só produza sarcasmos contra todos os Professores que defendem a prática da vacina os quais estão para os que a reprovam como 100 para 1 (Reflexões..., dez. 1811, p.181).

Em relação às moléstias que poderiam ser causadas pela vacina, os redatores afirmaram que não se tratava de fatos comprovados, mas apenas de especulações feitas por Heliodoro Carneiro. Os autores alegaram ainda que, pelo contrário, a vacina estaria curando “moléstias constitucionais”. Além disso, consideraram ridícula a preocupação de que, com o passar do tempo, pudesse ocorrer a transferência de “estranhas mudanças de simpatia quadrúpedes” (Reflexões..., dez. 1811, p.187) para os seres humanos, como haviam sugerido o próprio Carneiro e Moseley.

Segundo os redatores de *O Investigador Português em Inglaterra*, Heliodoro Carneiro, por não poder negar a eficácia da vacina, colocava em dúvida se a proteção seria contínua e se a vacina poderia causar doenças ou mortes após um longo período. Sobre essa questão, os autores reforçaram seus argumentos com base em observações e testes empíricos. Assim, na segunda parte da matéria, os autores apresentaram diversas evidências com o objetivo de destacar a eficácia da vacina e averiguar se ela poderia, de fato, causar novas doenças. Recorreu-se também ao argumento de autoridade, com a citação de vários médicos portugueses, muitos deles ligados ao alto escalão do governo, que, após a publicação da obra de Jenner, passaram a adotar a vacinação e se mostravam amplamente favoráveis ao método (Continuação..., 1812).

Uma dessas autoridades diretamente consultadas pelos autores foi Francisco José d’Almeida, médico da Real Câmara, que praticava a vacinação em Portugal havia pelo menos 12 anos e enviara uma carta aos redatores com suas observações. Na carta, publicada quase na íntegra, afirmou que nenhuma das pessoas por ele vacinadas havia contraído bexiga ou qualquer outra doença. O médico relatou ainda que, em toda a sua experiência com a vacina, apenas dois casos apresentaram sintomas mais graves: o da viscondessa de Fonte e o de seu filho, ambos com febres que quase resultaram em convulsões. No caso da viscondessa, a doença teria durado sete semanas (Continuação..., 1812).

Ainda nessa carta, Francisco José d’ Almeida teria mencionado alguns casos em que a vacina, além de proteger contra a varíola, trouxe uma melhoria geral na constituição das pessoas:

Era o viscondinho de Fonte-Arcada de mui débil entranhas, e tão sujeito a afecções de peito, que dava muito que temer a sua juventude. Visivelmente depois da Vacina perdeu aquela disposição catarrosa e hoje sadio passou mui bem pela escarlatina, e sarampo, o bofe parece são, e um jovem mui outro, do que foram em Menino, é este amável Fidalgo ... Sua Mãe que sofria debilidade de tubo intestinal, e todos os sintomas de Hipocondríase começada, melhorou sem outra Medicina ... Emilia Cambiaço ... era uma infante raquítica, de uma existência molesta, e que mal prometia sair da puerícia; vacinada, é hoje a mais vigorosa e encorpada de sua família (Continuação..., 1812, p.355).

Esses e outros casos teriam levado o médico a concluir que a modificação provocada pela vacina, na constituição humana, nunca era “maléfica”, mas sempre “salutífera”. Em relação à validade da proteção da vacina, o médico afirmou ter sido esta constatada, argumentando que, nas três graves epidemias de varíola que haviam ocorrido em Lisboa, naquele período, nenhum de seus vacinados havia adquirido a doença (Continuação..., 1812).

Outro importante relato médico, também apresentado nessa matéria, foi o de Bernardino António Gomes (1768-1823). Na opinião de Gomes, as crianças vacinadas tinham sintomas muito benignos, sendo inverossímil, então, afirmar que adquirissem outras enfermidades por causa da vacina. Mesmo nos casos que decorressem de um “vírus brutal”, este não produziria mais “degeneração na espécie humana” do que a que era causada pelos alimentos e por outros remédios retirados do reino animal. Ademais, para Gomes, por ocasião da última epidemia ocorrida em Lisboa, as pessoas variolizadas não ficaram protegidas contra a varíola, ao passo que as vacinadas, sim (Continuação..., 1812).

Além de relatos dos médicos, a matéria também apresentou os diversos casos de sucesso da vacinação ao redor do mundo, citando especialmente os casos da Inglaterra, país que seria emblemático dada a quantidade de dados que tinham sobre a vacinação. Para os redatores, a vacina seria evitada somente por aqueles que seguissem orientações erradas de alguns professores, como as do próprio Heliodoro Carneiro. Para dirimir as dúvidas remanescentes e refutar os autores ingleses mencionados por Carneiro, os redatores recomendaram a leitura de diversas obras publicadas na Inglaterra sobre a vacina, nas quais se travavam debates com os principais opositores da vacinação, muitos deles também citados por Heliodoro Carneiro. Isso evidencia não apenas a intensidade do debate no contexto inglês, mas também a forma como esses conhecimentos circulavam internacionalmente e exerciam influência em outras regiões, inclusive no Império português (Continuação..., 1812).

Por fim, a matéria corroborava o que havia sido constatado por estudiosos em várias instituições vacínicas pelo mundo e que não seria alterado pelos “tórridos interesses” de Heliodoro Carneiro. Para os redatores, os vacinadores seriam movidos pela “mais pura filantropia e crédito da arte de curar”. Os articulistas citaram ainda algumas considerações e dados das instituições vacínicas das cidades inglesas de Nottingham, Dublin, Edimburgo, Glasgow e Gloucestershire. A partir dos resultados apresentados por essas instituições, como a diminuição do número de mortes por varíola, os redatores procuravam demonstrar que a vacina era efetiva e que não estava transmitindo novas e perigosas doenças, como Carneiro havia afirmado em sua obra (Continuação..., 1812).

É importante ressaltarmos que o debate entre Heliodoro Carneiro e os médicos portugueses também deve ser entendido no contexto médico da época, no qual Carneiro, segundo o pesquisador Carlos Subtil (2017), integrava a classe de médicos conhecidos como os “médicos velhos”, que se apoiavam nos ensinamentos de Hipócrates, Galeno e Boerhaave e, com isso, desconfiava das inovações científicas que poderiam advir, por exemplo, da vacina e da utilização da química na medicina. Do outro lado, estariam os “médicos novos”, baseados em novas teorias, como Cullen, Brown, Pinel ou Broussais, que buscavam basear o tratamento e o conhecimento médico em novas evidências e conceitos, valorizando a inovação e o método científico (Subtil, 2017, p.54-57).

A questão da vacina evidenciava as tensões entre as duas categorias de médicos em Portugal: os “médicos velhos”, que se apegavam a métodos tradicionais, e os “médicos novos”, influenciados pelas novas correntes científicas. A crítica aos primeiros centrava-se no fato de que, ao permanecerem presos a práticas ultrapassadas, acabavam por atrasar o progresso científico e social. Essa crítica ganha força no contexto do Iluminismo português, que, embora tardio em relação aos centros europeus, começou a se consolidar na segunda metade do século XVIII (Varela, 2006, p.51-52).

É interessante observar que, embora Heliodoro Carneiro se alinhasse aos chamados “médicos velhos”, sua formação ocorreu em um momento de transição intelectual, quando as “novas ideias” já vinham sendo implementadas na Universidade de Coimbra, que, após a Reforma Pombalina de 1772, passou a ocupar uma posição central na formação científica e médica do reino. Essa reforma, inserida no contexto do Iluminismo português, buscava modernizar o ensino superior em Portugal, substituindo o modelo escolástico por um currículo fundamentado nas ciências naturais e experimentais. No curso de Medicina, por exemplo, passaram a ser incluídas disciplinas como anatomia, fisiologia, química, botânica médica, história natural e, ainda que de forma incipiente, prática clínica. O novo modelo enfatizava o conhecimento empírico e a observação direta, aproximando-se dos paradigmas médicos modernos (Maiolini, 2023). No entanto, como demonstra a própria obra de Heliodoro Carneiro, a introdução dessas novas concepções não ocorreu sem resistências, revelando as tensões entre tradição e inovação no interior do saber médico oitocentista.

O Iluminismo em Portugal adquiriu um caráter marcadamente pragmático e utilitarista, no qual razão, natureza e prática deveriam se articular para promover o bem comum e a melhoria das condições materiais de vida da população. Com isso, o Estado passou a incentivar a formação de cientistas que, orientados pelas ideias ilustradas europeias, buscavam aplicar esse conhecimento à realidade local, tanto metropolitana quanto colonial, especialmente em áreas como comércio, indústria, artes e medicina. Essa adaptação das “receitas” europeias aos desafios portugueses refletia uma tentativa de modernização acelerada, impulsionada pelo saber científico (Dias, 1968). Nesse contexto, a prática da vacinação passou a ganhar crescente respaldo entre a maior parte da intelectualidade portuguesa, sobretudo entre os médicos formados sob a influência das reformas iluministas.

## Considerações finais

Heliodoro Jacinto de Araújo Carneiro, embora afastado de uma parcela de médicos portugueses favoráveis à vacina, não foi um pensador isolado, pois havia baseado seus argumentos em vários outros autores seus contemporâneos. Carneiro compartilhava do anseio de uma série de médicos que haviam proposto diversas críticas empíricas e teóricas à prática da vacinação na Inglaterra. Suas ideias também iam ao encontro de uma parcela da população que era refratária ao método.

Os argumentos contrários à vacina apresentados por Carneiro eram plausíveis dentro do campo teórico da medicina da época e não foram necessariamente vencidos nesse campo, mas “derrotados” no campo prático, quando a empiria acabou se tornando o “juiz” da vacina, que logrou êxito por sua enorme eficácia. Assim, a prática, ainda que cercada de polêmicas e controvérsias, institucionalizou-se e manteve continuidade no Brasil e em Portugal.

Embora as ideias de Carneiro fossem plausíveis, elas contavam com poucos adeptos no campo médico e intelectual do Império. A posição social que ele ocupava, aliada ao desejo de se aproximar da corte portuguesa, de obter cargos governamentais e de estabelecer relações com os intelectuais da época, contribuiu para que esse médico mudasse sua postura em relação à vacina.

Por fim, no debate médico da época, surgiram diversos “exemplos de exceção”, nos quais ambos os lados traziam efeitos poucos críveis de serem atrelados à vacinação, tanto de curas milagrosas quanto de mazelas graves. Assim, ressaltando o caráter coletivo da atividade científica, destaca-se o expressivo grupo de médicos que praticavam a vacinação em várias partes do mundo, registrando os sintomas e realizando experimentações que contribuíram para a validação da vacina antivariólica. Esse foi um processo que se estendeu por várias décadas, pois, mesmo após sua institucionalização, essa prática médica continuou sendo alvo de controvérsias, tanto no campo científico quanto no social. A história da vacina antivariólica evidencia que as práticas médicas demandam não apenas validação científica, mas também legitimidade social, uma vez que, mesmo sendo eficazes, estão inseridas em disputas e negociações que extrapolam o campo técnico.

## Data Availability

Não estão em repositório.

## NOTAS

1 É importante ressaltar que a obra de Edward Jenner *An inquiry into the causes and effects of the variolæ vaccinæ*, de 1798, foi traduzida para o português somente em 1803, com o título *Indagação sobre as causas, e efeitos das bexigas de vaca, moléstia descoberta em alguns dos condados ocidentais da Inglaterra, particularmente na comarca de Gloucester, e conhecida pelo nome de vacina*.

2 Heliodoro Jacinto de Araújo Carneiro formou-se em medicina, em 1798, na Universidade de Coimbra, e passou a maior parte de sua vida fora de Portugal, empregado primeiramente em comissões científicas e posteriormente diplomáticas. Recebeu o título de visconde de Condeixa por serviços prestados. Ver o verbete Heliodoro Jacinto de Araújo Carneiro em Silva (1840).

3 Na Grã-Bretanha, John Bull era a personificação de um homem forte como um touro, ideia criada, em 1712, por John Arbuthnot, médico, satírico e sábio.

4 As vacinas “espúrias” seriam variedades do imunizante que ocorriam quando ele, em vez da doença normal, fazia surgir somente esponjas ou verrugas no lugar inoculado, ou mesmo formas similares da reação vacinal.

5
*O Investigador Português em Inglaterra ou Jornal literário, político etc.* tinha como redatores os médicos Bernardo José de Abrantes e Castro, Vicente Pedro Nolasco da Cunha e Miguel Caetano de Castro. Embora fosse patrocinado pela Coroa portuguesa, era publicado em Londres. Circulou entre julho de 1811 e fevereiro de 1819. A referida matéria tem sua continuação no mesmo periódico, em sua edição de 1812 (Continuação..., 1812).
